# Green-Synthesized Silver Nanoparticles: Antifungal and Cytotoxic Potential for Further Dental Applications

**DOI:** 10.3390/jfb14070379

**Published:** 2023-07-20

**Authors:** Widadh Klein, Enas Ismail, Ernest Maboza, Ahmed A. Hussein, Razia Z. Adam

**Affiliations:** 1 Prosthodontics Department, Faculty of Dentistry, University of the Western Cape, Parow, Cape Town 7505, South Africa; 2 Physics Department, Faculty of Science (Girl’s Branch), Al Azhar University, Cairo 11884, Egypt; 3 Department of Chemistry, Cape Peninsula University of Technology, Bellville 7535, South Africa

**Keywords:** silver nanoparticles, green synthesis, *Berzelia lanuginose*, *Helichrysum cymosum*, *Searsia crenata*, *Candida albicans*, antifungal, cytotoxicity

## Abstract

Fungal infections caused by *Candida albicans* (*C. albicans*) are one of the most prevalent types of oral disorders in the elderly. It has been reported that drug resistance to fungal pathogens poses a severe risk to global healthcare systems and public health. Therefore, the goal of this work is to investigate the cytotoxic and antifungal properties of silver nanoparticles (AgNPs) produced using three different natural extracts: *Berzelia lanuginose*, *Helichrysum cymosum*, and *Searsia crenata*. According to the UV-Vis results, the synthesized AgNPs via *B. lanuginose*, *H. cymosum*, and *S. crenata* show surface plasmonic resonance (SPR) peaks at 430, 440, and 428 nm, respectively. HR-TEM revealed different shapes for the nanoparticles within the size ranges of 16–20, 31–60, and 57–72 nm for *B. lanuginose*, *H. cymosum*, and *S. crenata*, respectively. Using a human oral fibroblast cell line, the cytotoxicity of both AgNPs and plant extracts was tested at concentrations of 0.007, 0.012, 0.025, and 0.062 mg/mL (buccal mucosa fibroblasts). The antifungal activity showed growth inhibition zones of approximately 18 mm, 18.67 mm, and 18.33 mm for the AgNPs conjugated with *B. lanuginose*, *H. cymosum*, and *S. crenata,* respectively. For the studied samples, the minimum inhibitory concentration (MIC_50_) was less than 0.015 mg/mL. The AgNPs exhibited antifungal activity that was concentration- and size-dependent. The results of this study offer new insights into the cytotoxicity and antifungal activity of the green-synthesized AgNPs.

## 1. Introduction

Nanoparticles have been utilized in dental applications to increase therapy effectiveness, shorten treatment times, and lower the possibility of adverse effects. Since the beginning of the millennium, nanoparticles have been used in dental applications. Initially, dental materials like fillings and crowns were made tougher and more resilient using nanoparticle technology. Nanoparticle applications have grown over time to incorporate tooth whitening, medicine administration, and other medical procedures. In recent years, therapies for periodontal disease, tooth decay, and other dental issues have become more effective due to the use of nanoparticles. According to Tsakos et al., the population is aging more rapidly than in the past [1]. This would indicate that the average age of patients wearing removable dental prostheses is rising and that there is a higher risk of developing oral diseases [2]. Dental care for the elderly should be focused on their daily lives, such as on denture cleaning, wearing dentures, and tooth loss. The presence of a prosthesis can alter the oral environment, which can lead to fungal infections such as denture stomatitis [3]. Denture stomatitis, a disorder that causes inflammation of the mouth and gums, is brought on by a species of fungus called *Candida albicans (C. albicans)*. *C. albicans* is a normal oral commensal. It is known for its ability to induce systemic and superficial infections in human hosts. Fungal pathogens can persist inside the host due to the development of multidrug resistance characteristics and pathogenicity, which often leads to the failure of therapeutic strategies [4,5]. Several topical and systemic treatments are available for denture stomatitis [6]. Drug resistance to fungal diseases is becoming a serious concern in global healthcare and public health systems. In the presence of fungal diseases that are multidrug-resistant, it is crucial to create new antifungal medications. Medically important plants can be integrated into the drug delivery process by using the knowledge of plants as medicines, which might then be used to help against antimicrobial resistance. This creates the need for further exploration of alternative antifungal therapies, such as silver nanoparticles (AgNPs) [7]. It is commonly known that AgNPs have long-term antibacterial and antifungal activity, low bacterial resistance, low toxicity, and sustained ion release [8]. AgNPs have been synthesized using a variety of synthesis techniques [9]. Different conventional techniques were used for the NP synthesis process, which revealed costly, dangerous substances. Hence, this has a negative impact on the toxicity of the synthesized NPs. The conventional techniques are commonly known as physical or chemical methods, such as chemical/physical vapor deposition, the reduction method, sol-gel processing, and hydrothermal synthesis. On the other hand, green synthesis has several advantages that are consistent with green chemistry principles. For example, plant materials that are commonly available, accessible, and cheap are used. Plants contain different components that can be employed to reduce and stabilize nanoparticles without the use of external stabilizers. The solvents used are typically eco-friendly, protecting the environment from dangerous chemical residues, and the synthesized NPs are substantially less toxic [9,10]. AgNPs that have been greenly synthesized exhibit good yields, stability, and solubilities. Well-defined size and shape can also be achieved using green approaches [10]. Many plants have been used in the green synthesis of AgNPs [11,12,13,14]. In particular, *H. cymosum* has biomedical properties, such as antibacterial, antioxidant, antifungal, antiviral, anti-HIV, and anti-inflammatory properties [15]. *B. lanuginose and S. crenata* are medicinal plants that have been utilized in traditional medicine for many years and are still widely used today [16]. In this study, a green synthesis process for silver nanoparticles using extracts from *B. lanuginose*, *H. cymosum*, and *S. crenata* was carried out. Antifungal properties of the obtained AgNPs were studied against *C. albicans* (ATCC 90028), as well as their cytotoxic activity.

## 2. Materials and Methods

### 2.1. Extraction and Purification of the Natural Extracts

Aerial parts of three plant species, namely *B. lanuginose*, *H. cymosum*, and *S. crenata,* were collected and identified from two neighboring sites within the Western Cape Province, namely the University of the Western Cape Reserve and the Cape Peninsula University of Technology campus in Bellville. After allowing the plant leaves to dry, they were ground up and extracted with water (50 mL of distilled water added to 5 g of each plant powder) at 70 °C for 15 min. The extracts were filtered and then centrifuged at 3750 rpm for two hours using the Allegra^®^ X-12R centrifuge (Beckman Coulter, Cape Town, South Africa). Each extract’s supernatant was then filtered via 0.45 m filters. The extracts were then freeze-dried with a FreeZone 2.5 L freeze dryer after being maintained overnight (~12 h) at −80 °C (Labconco, Kansas City, MO, USA) [17].

### 2.2. Biosynthesis of Silver Nanoparticles

Silver nitrate was purchased from Sigma Aldrich (Cape Town, South Africa). A quantity of 250 mL of 1 mM silver salt was added to 50 mL stock solutions of the three tested plant extracts with escalating concentrations (0.007 to 16 mg/mL). At a shaking rate of 40 rpm, the plates were incubated at 70 °C for one hour. The abbreviations Bl.AgNPs, Hc.AgNPs, and Sc.AgNPs refer to the green-synthesized AgNP samples from *B. lanuginose*, *H. cymosum*, and *S. crenata*, respectively.

### 2.3. UV-Visible Spectroscopic Analysis

The UV-vis spectrophotometer SPECTROstar Nano 2450 (BMG LABTECH, Ortenberg, Germany) was used to measure the absorption bands resulting from electrons confined on the biosynthesized silver particles. The stability of the synthesized AgNPs was assessed using the same UV-Vis spectrophotometer by incubating the AgNPs with 0.5% bovine serum albumin (BSA) and 0.5% cysteine. By observing the changes in UV-Vis spectra after 2 h, 4 h, 6 h, 12 h, and 24 h, the stability of the AgNPs was ascertained.

### 2.4. HRTEM Analysis 

The morphology and crystallinity of the AgNPs were examined using high-resolution transmission electron microscopy (FEI Tecnai G2 F20 S-Twin HRTEM, operating at 200 kV). A histogram of the nanoparticle sizes was obtained using Image J (1.44) software.

### 2.5. Dynamic Light Scattering Analysis 

The hydrodynamic size, polydispersity index, and zeta potential of the AgNPs were measured using a Malvern Zetasizer instrument (Malvern Ltd., Malvern, UK) operating at angles of 25 and 90 degrees. After cooling to room temperature, solutions of AgNP biosynthesized samples were measured in disposable quartz cuvettes [10].

### 2.6. Antifungal Activity/Assay

The UWC Oral and Dental Research Laboratory provided *C. albicans* (ATCC 90028). This was made following the accepted procedure (CLSI, 2017) to check for any antifungal activity from the biosynthesized AgNP samples and to assess the most effective sample on sessile yeast. Two tests were conducted to determine the effect of the AgNPs on *C. albicans*: (a) Kirby–Bauer test measuring zones of inhibition, and (b) minimum inhibitory concentrations (MIC) using a cell viability and proliferation XTT assay.

Filter discs of 9 mm in diameter (FN30, Lasec, Cape Town, South Africa) were used to determine the zones of inhibition. The discs were infused with the biosynthesized AgNPs and 0.2% chlorhexidine as the positive control. Kirby–Bauer was applied on 100 µL of 0.5 McFarland standard (McF) *C. albicans* spread on Mueller–Hinton (MH) agar and incubated at 37 °C for 24 h (CLSI, 2017). The XTT assay was used according to the manufacturer’s guidelines (Sigma–Aldrich Company Ltd., Gillingham, Dorset, UK) with minor changes on 150 µL (0.5 McF) *C. albicans*.

Four-time intervals at 4, 6, 24, and 48 h were used to examine each sample of the biosynthesized AgNPs in triplicate. The AgNP: *C. albicans* ratio was 1:1 with 50 µL BHI added [18]. After the set incubation period, the plates were washed to eliminate any remaining planktonic yeast. Subsequently, 50 mL of the XTT labelling reagent and 100 mL of MH broth were added to the wells with sessile yeast. After the predetermined incubation period appropriate for a 96-well plate format, MICs were read using a plate reader (Biocom model SMR 16.1) at 492 nm. 

### 2.7. Cytotoxicity Testing

Human buccal mucosa fibroblasts, obtained from the Oral and Dental Research Laboratory at the University of the Western Cape, were utilized in the MTT assay to investigate the potential cytotoxicity of the extracts by mimicking the oral environment [19]. MTT tests were conducted according to the manufacturer’s instructions. AgNPs were examined in three samples at concentrations of 0.007, 0.012, 0.025, and 0.062 mg/mL. Cell survival was determined using an RT2100C spectrophotometer at 542 nm. All the antifungal and cytotoxic tests were analysed in triplicate for the accuracy and reproducibility of the results. 

### 2.8. Statistical Analyses 

Stata statistical software release 15 (College Station, TX, USA: Stata Corp LLC) was used to analyze the data. All tests were deemed statistically significant at *p* < 0.05. This was a double-blinded study, as the statistician did not know which sample yielded the specific result as the results for the samples were lettered instead of named.

## 3. Results and Discussion

### 3.1. UV-Vis Spectroscopy Analysis

**Figure 1:**
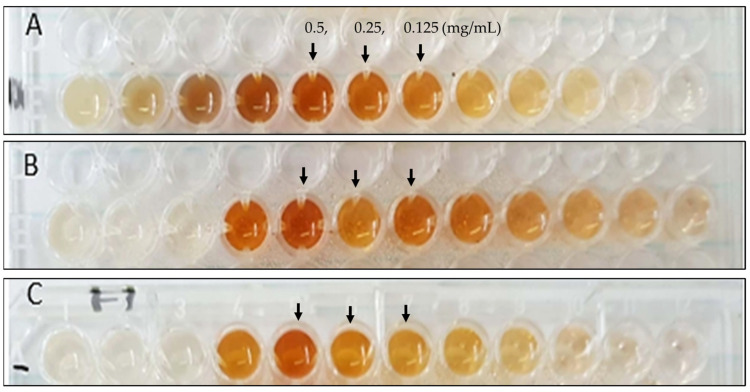
The biosynthesis process for AgNPs using natural extracts of (**A**) *B. lanuginose;* (**B**) *H. cymosum*; and (**C**) *S. crenata* natural extracts. Three different concentrations were selected for further analysis.

**Figure 2:**
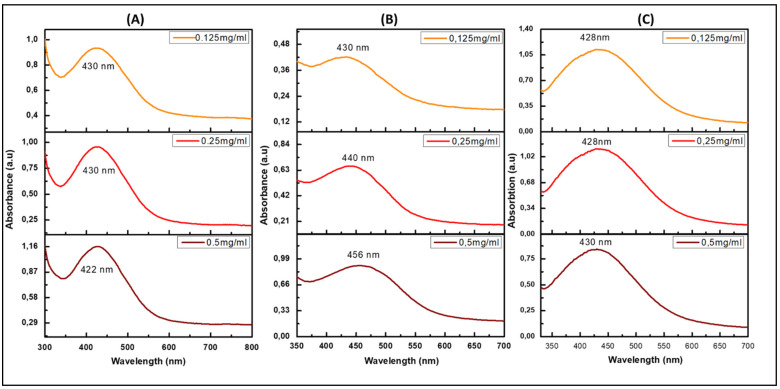
The UV-Vis absorption spectra for the green-synthesized AgNP samples using three natural extracts, namely, (**A**) Bl.AgNPs; (**B**) Hc.AgNPs, and (**C**) Sc.AgNPs at the three selected concentrations.

For the green synthesis of AgNPs, three distinct leaf extracts (*B. lanuginose*, *H. cymosum*, and *S. crenata*) were used. As a precursor for the synthesis process, silver nitrate revealed a fast conversion into AgNPs, which was demonstrated by noticeable color changes from clear to yellow/dark brownish yellow, as seen in Figure 1. As a phase in the optimization of silver biosynthesis, several serial dilutions of the extracts were used and incubated at 70 °C. Figure 1 illustrates several sample colors that show the direction of initial identification of the optimal plant concentration for the green synthesis of AgNPs. The three selected plant extracts were evaluated in the screening procedure at different concentrations. Promising samples were considered for further characterization. The formation of AgNPs was verified by absorbance measurements using UV spectroscopy. The findings demonstrate the characteristic surface plasmon resonance (SPR) of the green-synthesized AgNPs, illustrating the size and shape of the nanoparticles affected the SPR spectra [20]. The UV-vis spectra for the green-synthesized AgNP samples using extracts of *B. lanuginose, H. cymosum, and S. crenata* are shown in Figure 2. Figure 2A reveals different absorbance bands at 422, 430, and 430 nm correlated to the synthesized AgNPs (Bl.AgNPs) using different concentrations of *B. lanuginose* extract (0.5, 0.25, and 0.125 mg/mL). Meanwhile, for the Hc.AgNP sample, Figure 2B depicts UV spectra bands at 456, 440, and 430 nm, correlated to the different concentrations of *H. cymosum* extracts (0.5, 0.25, and 0.125 mg/mL, respectively). In Figure 2C, the SPR correlated to the Sc.AgNP sample revealed different absorbance bands at 430, 428, and 428 nm, corresponding to the *S. crenata* extract concentrations (0.5, 0.25, and 0.125 mg/mL, respectively). The UV results revealed absorbance bands within the range of 400–450 nm characteristic of the formation of AgNPs [21,22,23]. Changes in color and the presence of SPR confirmed the formation of AgNPs [24]. The results indicate the vital effects of *B. lanuginose, H. cymosum, and S. crenata* extracts as reducing agents. 

### 3.2. Stability Testing of the Synthesized AgNPs

In this report, the green-synthesized AgNPs are evaluated for further biomedical applications. Hence, it is important to evaluate their stability in different aqueous buffer solutions. Two different media, cysteine and bovine serum albumin (BSA), were selected for this study. In general, the effectiveness of nanoparticles depends on their stability over a certain period and in different biological solutions [25]. The stability of the AgNPs was recorded by UV-vis spectra at 2, 4, 6, 12, and 24 h using two different media, namely cysteine and BSA. 

**Figure 3:**
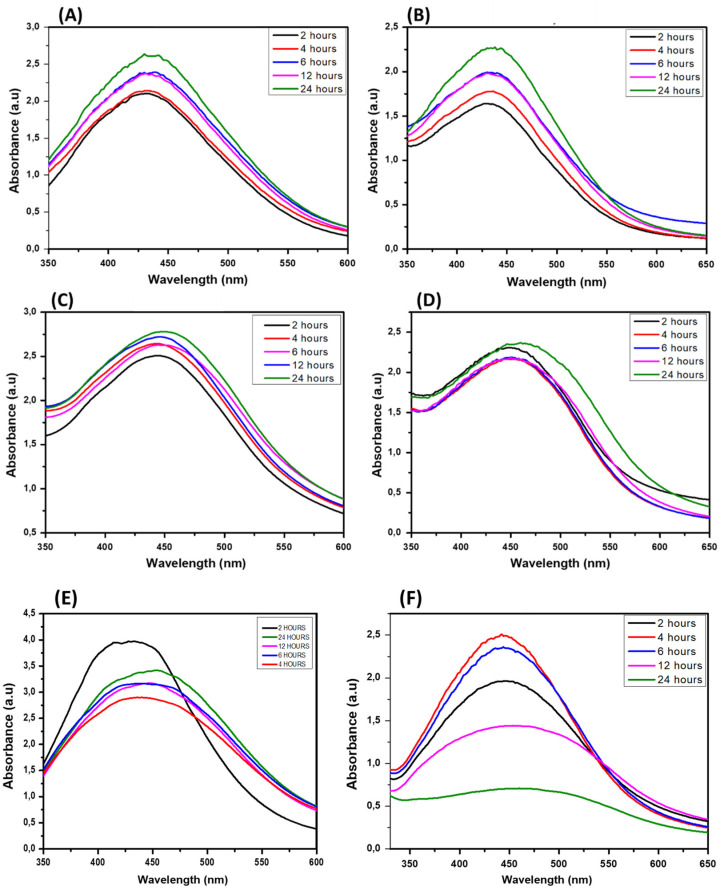
UV-Vis absorption results representing the stability investigation studies of (**A**,**B**) Bl.AgNPs; (**C**,**D**) Hc.AgNPs; and (**E**,**F**) Sc.AgNPs using BSA and cysteine, respectively, after 24 h.

Figure 3 represents the stability test results for Bl.AgNP, Hc.AgNP, and Sc.AgNP samples. The biological stability of the green-synthesized AgNPs in Bl.AgNPs and Hc.AgNPs is depicted with a minimal change in their corresponding UV-Vis spectra in the BSA and cysteine mediums. Sc.AgNPs indicated a moderate shift in the UV-Vis spectra with BSA medium (Figure 3E). Furthermore, the Sc.AgNP sample stability results in cysteine showed a broadening and decrease in the intensity of the characteristic UV spectra of the synthesized AgNPs (Figure 3F). This may be an indication of the formation of larger particles [26]. The stability results confirmed the vital effects of *B. lanuginose* and *H. cymosum* as effective capping agents. According to Badeggi (2020), BSA has about 50 lysines on its surface, giving it a high affinity for negative NP surfaces [9]. Hence, a further DLS analysis investigation was carried out to confirm the negative ZP values of the green-synthesized AgNPs.

### 3.3. Dynamic Light Scattering (DLS) 

**Figure 4:**
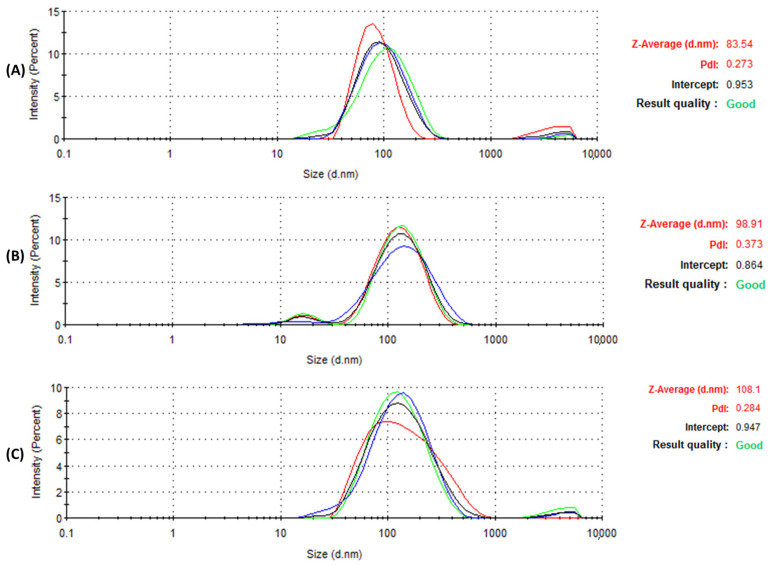
DLS analysis representing the size distribution and PDI of the green-synthesized AgNP samples; (**A**) Bl.AgNPs; (**B**) Hc.AgNPs; and (**C**) Sc.AgNPs.

DLS is a technique used to measure the size distribution of AgNPs. For the consistency of measurements, the reproducibility concept was used for each sample to ensure obtaining the same result when measuring repeatedly under the same conditions. The average result is represented in Table 1. The DLS results, represented in Table 1 and Figure 4, revealed an average size distribution of 83.54, 98.91, and 108.1 nm corresponding to Bl.AgNPs, Hc.AgNPs, and Sc.AgNPs, respectively. The represented size by the DLS is a hydrodynamic size that represents the metallic core and the attached biomolecules [12]. Figure 4 also represents the polydispersity index (PDI) for the tested green-synthesized AgNP samples. PDI is a helpful indicator for evaluating the NPs’ size distribution and determining the particle uniformity. The PDI results reflect values of 0.27, 0.37, and 0.28 corresponding to the biosynthesized AgNP samples: Bl.AgNPs, Hc.AgNPs and Sc.AgNPs, respectively. The International Standards Organization (ISO) indicate that monodisperse samples are more likely to have polydispersity index values below 0.05, whereas polydisperse samples are more likely to have values above 0.7 [27]. Hence, Bl.AgNPs and Sc.AgNPs are more likely to have a uniform shape due to their low PDI values. Hc.AgNPs had a slightly higher PDI value of 0.37, indicating the possibility of polydispersity. Hence, the three tested AgNP samples (Bl.AgNPs, Hc.AgNPs, and Sc.AgNPs) were considered for further HRTEM investigation.

**Figure 5:**
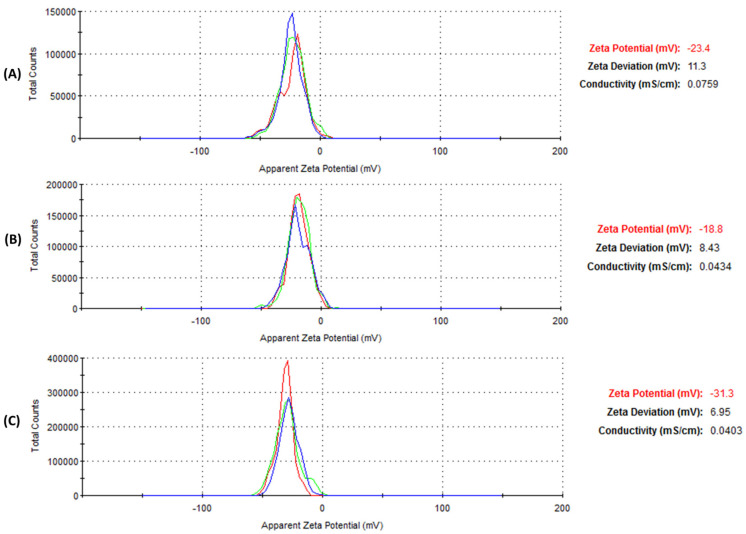
Zeta potential results for the green-synthesized AgNP samples: (**A**) Bl.AgNPs; (**B**) Hc.AgNPs; and (**C**) Sc.AgNPs, respectively.

Zeta potential tests were conducted to assess the stability, as seen in Figure 5A–C. Table 1 represents the ZP values of −23.4, −18.8, and −31.3 mV obtained for Bl.AgNPs, Hc.AgNPs, and Sc.AgNPs, respectively. The AgNPs’ negative zeta-potential values are within the normal range for stable colloidal dispersions [25]. Natural extract biomolecules on the surface of the biosynthesized AgNPs may be responsible for the negative zeta potential values. This may lead to a repulsive force between AgNPs, resulting in less aggregation [12]. Hence, the negative ZP values also predict the long-term stability of the synthesized AgNPs in solution. The DLS data agrees with the stability results. As a result, the interaction between the synthesized AgNPs and BSA is strengthened. 

### 3.4. HRTEM Analysis

**Figure 6:**
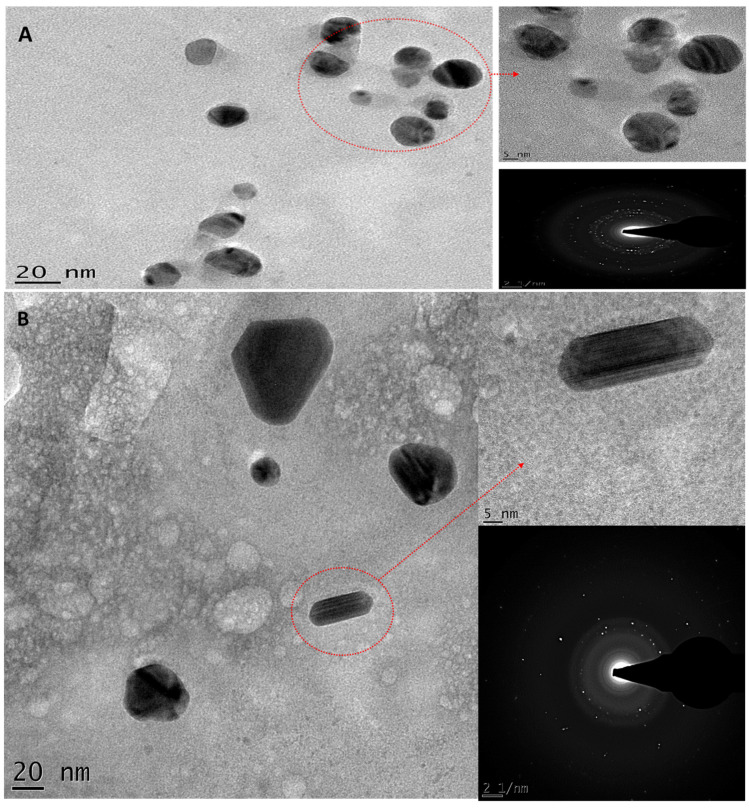

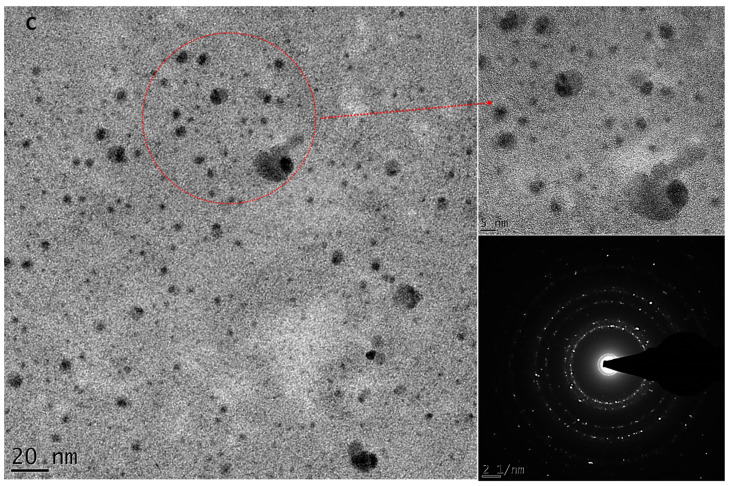
The HRTEM results and the SAED pattern for the green-synthesized AgNP samples: (**A**) Bl.AgNPs; (**B**) Hc.AgNPs; and (**C**) Sc.AgNPs.

High-resolution transmission electron microscopy (HRTEM) [24] is an advanced technique for evaluating the morphology, distribution, and crystallinity of NPs. Figure 6 shows the HRTEM results and the selected-area electron diffraction (SEAD) patterns of the AgNP (Bl.AgNPs, Hc.AgNPs and Sc.AgNP) samples synthesized by bio-reduction of Ag+ using extracts of *B. lanuginose*, *H. cymosum*, and *S. crenata*, respectively. The majority of the synthesized AgNP samples from *B. lanuginose* and *S. crenata* extracts were spherical in shape (Figure 6A,C). The spherical shapes of AgNPs are a common feature [28,29]. The two samples also revealed well-distributed AgNPs with no aggregations, confirming *B. lanuginose* and *S. crenata* extracts as reducing and capping agents. Interestingly, green-synthesized AgNPs using *H. cymosum* extracts (Figure 6B) revealed a variety of shapes and sizes. Rectangular, pentagonal, and spherical shapes were detected [30]. The loss of protective biomolecules, which help maintain the homogeneity of the shape during growth, is thought to be the cause of this shape anisotropy. This forces them to develop other shapes, like pentagons and hexagons, to achieve thermodynamic stability [31]. These nanoparticles’ monodispersity is achieved by the polyphenols capping layer, confirming the vital effect of the extract as a capping agent [10]. It is also reported that polyphenols in the extracts enhance the reduction of Ag^+^ to Ag^0^, revealing the vital effect of the extract as a reducing agent. The oxidized polyphenols attach to the AgNPs and simultaneously stabilize them [10,32].

The selected-area electron diffraction (SAED) patterns for the synthesized AgNP samples revealed the crystalline nature of the green-biosynthesized AgNPs at 70 °C. The results revealed a diffraction ring pattern that may be attributed to the face-centered cubic (fcc) structure. Figure 6C represents four diffraction rings associated with the (111), (200), (220), and (311) lattice fcc planes. This confirms the biosynthesized material’s high degree of crystallinity for Sc.AgNPs [33]. The AgNP’s crystallinity with Bl.AgNPs and Hc.AgNPs is demonstrated by the concentric rings observed in their SAED pattern (Figure 6A,B). It is noticed that the crystallinities of the Sc.AgNP samples were the highest (Figure 6C). This may be attributed to the effect of the extract on the NP’s crystallinity degree.

### 3.5. Antimicrobial Testing Results

#### 3.5.1. Modified Kirby–Bauer Assay on *C. albicans*

**Figure 7:**
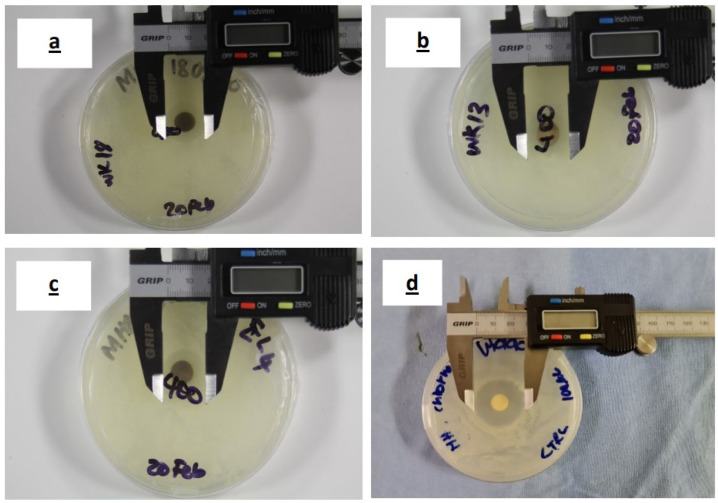
Inhibition zones for the green-biosynthesized AgNP samples: (**a**) Bl.AgNPs; (**b**) Hc.AgNPs; and (**c**) Sc.AgNPs. (**d**) Zone of inhibition for 0.2% chlorhexidine.

The inhibition zones on agar plates and the calculation of MIC values on microplates were used to assess the antifungal activity of the aqueous plant extracts and AgNP samples (Bl.AgNPs, Hc.AgNPs and Sc.AgNPs) against *C. albicans*. All AgNPs displayed antifungal efficacy with comparatively similar zones of inhibition. The Kirby–Bauer test revealed that AgNP samples were more efficient against *C. albicans* than their corresponding aqueous extracts. Figure 7a–d represents the zones of inhibition for Bl.AgNP, Hc.AgNP and Sc.AgNP samples with ranges 17–19, 18–19, and 18–19 mm, respectively. The inhibition zone for 0.2% chlorhexidine (positive control) was in the range 26–28 mm. Hence, the conventional zone of inhibition test confirmed the antibacterial response of the green-synthesized AgNP samples [34].

#### 3.5.2. XTT: Minimum Inhibitory Concentration for AgNPs

Minimum inhibitory concentration (MIC) was calculated to determine the effect of concentration on *C. albicans* over time. OD values were taken for various AgNP concentrations of 0.125, 0.062, 0.025, 0.0125, 0.007, 0.003, and 0.001 mg/mL. Four defined intervals (i.e., T1—4 h, T2—6 h, T3—24 h, and T4—48 h) were applied for the OD measurements. A 90% growth reduction occurred between 0.125 and 0.062 mg/mL for all the biosynthesized AgNP samples at 4 h. MIC_50_ has previously been satisfactorily demonstrated using the XTT assay [35]. The XTT reduction was visually observed for color change (from clear to yellow and finally to red) to predict the possible MIC_50_ values for the respective AgNPs. The OD readings are shown below in Table 2.

**Figure 8:**
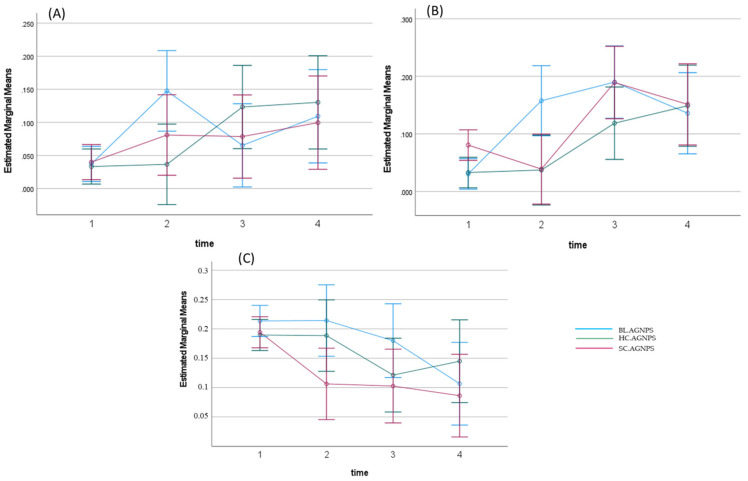
The estimated marginal means of the OD, defining the limits within which the respective MIC_50_ values lie for each biosynthesized AgNP sample at various concentrations over time: (**A**) 0.015 mg/mL, (**B**) 0.007 mg/mL, (**C**) 0.0039 mg/mL.

The corresponding estimated marginal means of the OD on the yeast suggest the limits encompassing the MIC_50_ values for the three biosynthesized AgNP samples as shown in Figure 8. Figure 8A–C represents the different concs. (0.015, 0.007 and 0.003) of the biosynthesized AgNP samples (Bl.AgNPs, Hc.AgNPs, and Sc.AgNPs) at different time intervals (4, 6, 24, and 48 h, respectively). The results indicate a reduction in biofilm growth for all biosynthesized AgNP samples compared with the untreated control group at all time points. Qualitative observation of the MIC_50_ for all samples seemed below the conc. 0.015 mg/mL after 48 h.

The main concentrations of all three treatments inhibited biofilm growth rates, while growth below the concentration 0.0025 mg/mL was profuse, comparable with untreated biofilms. All biosynthesized AgNP samples showed a 90% growth reduction between 0.125 and 0.06 mg/mL after 4 h. Percentage reduction was calculated from the formula below:

$$\%OD\;reduction=\frac{OD\;sample\;treated-OD\;sample\;untreated}{OD\;sample\;untreated}\times 100$$

The average OD readings for the untreated biofilms were used as a reference. The calculated values were then used to model average changes in biofilm survival after treatment at different concentrations observed at different time intervals. Regression analysis was thereafter used to calculate the respective MIC_50_ (Table 3). The linear regression analysis formula for the 4 h interval was as follows: y = −14.029x + 92.461; R² = 0.7767 samples; y = −14.372x + 89.375; R² = 0.7124; and y = −12.264x + 79.751; R² = 0.7726, for HcAgNPs, Bl.AgNPs, and Sc.AgNPs.

The calculated MIC_50_ values were within the observed range of color change, suggesting a 50% reduction in the yeast’s metabolic activity, that is, between the fourth and the fifth two-fold dilutions from 0.25 mg/mL of the biosynthesized AgNPs after the first 4-hour interval. This was consistent with the estimated marginal means of the OD readings, where the observed color change suggested upper and lower limits that included MIC_50_. The calculated MIC_50_ values from the Sc.AgNP and Hc.AgNP samples, represented in Table 3, were above the upper limit (6.25%). In comparison with the other two plants, Bl.AgNP’s calculated MIC_50_ was consistently about 6,2% throughout the first two intervals. The Sc.AgNP sample after the second interval (6 h) indicated a weaker effect, with a MIC_50_ of 10.32%, in comparison with Bl.AgNPs and Hc.AgNPs at 6.22 and 6.32%, respectively. The effects of all biosynthesized AgNPs were similar, with a weaker effect from Bl.AgNPs at 10 and 20% for MIC_50_.

These results suggest that the green-biosynthesized AgNPs from the three plant extracts inhibited the growth of the yeast, albeit at low concentrations. Hc.AgNPs demonstrated the highest overall potency over 24 h, while Bl.AgNPs showed lowered potential after 24 h. These nanoparticles, however, may prove instrumental in controlling the rampant growth of *C. albicans* without disrupting the normal flora of the mouth, by completely eradicating the yeast.

### 3.6. Cytotoxicity

In this study, cytotoxicity to a buccal mucosa cell line was evaluated quantitatively using MTT assay according to EN ISO10993-5 with a reduction in cell viability of less than 30% considered a cytotoxic effect. The first cell line used was a human oral fibroblast cell line established in the Oral and Dental Research Laboratory, University of the Western Cape, as these fibroblasts were well suited for the oral environment. Stocks of these cells were kept frozen in liquid nitrogen and retrieved for use. Cells were maintained and cultured in standard conditions. Overall, all plant extracts (*B. lanuginose, H. cymosum*, and *S. crenata*) did not demonstrate any cytotoxic effects, as represented in Table 4.

The biosynthesized AgNPs had toxicity effects above 0.025 mg/mL concentrations, except for the Sc.AgNP samples, which had 39,4% cell survival at a 0.025 mg/mL treatment concentration (Table 4). This is consistent with the stability test results. Each biosynthesized AgNP sample had statistically significant cytotoxicity results compared with its corresponding plant extract, across all concentrations. This indicates that the use of these plant extracts as capping agents increased the cytotoxic effect, albeit only noticeably at concentrations above 0.062 mg/mL. When observing the increase in the concentrations of the AgNPs, toxicity was concentration-dependent, with lower concentrations displaying higher cell survival. AgNP samples demonstrated 40.32, 30.48, and 25.27% cell survival rates for Bl.AgNPs, Hc.AgNPs, and Sc.AgNPs, respectively, at a 0.062 mg/mL concentration. This suggests that the Sc.AgNP samples could be more toxic than the other two biosynthesized samples, with Bl.AgNPs having the highest cell survival, i.e., the lowest toxicity. This point analysis was used because of its proximity to the above-defined toxicity (i.e., <30% survival). However, it is noticed that Hc.AgNP samples had the highest cell survival rates at concentrations below 0.037 mg/mL, suggesting the lowest toxicity of the three AgNP samples.

Numerous studies have shown that the particle sizes of AgNPs affect their cytotoxicity. Some of these investigations concluded that AgNPs’ cytotoxicity increases with decreasing particle size [36,37,38,39]. AgNPs have demonstrated a critical impact on cell survival and the production of reactive oxygen species and lactate dehydrogenase activity in a size-dependent way, across several cell lines [36]. Bl.AgNPs revealed the highest cell death rates. According to Paknejadi et al. (2018), small nano-sized silver particles may have cytotoxic effects on normal cells, especially at prolonged exposure times and high concentrations [40,41].

## 4. Conclusions

The green synthesis approach for metallic nanoparticles is an important research area that has received a lot of attention in recent years. This is attributed to their potential positive environmental and economic impact. The present state of the art in the green synthesis of AgNPs involves the utilization of numerous natural sources, such as plant extracts, bacteria, fungi, and others. Green synthesis processes using plant extracts are often less expensive and less harmful to the environment than standard chemical procedures.

Their potential for use in the management of *C. albicans* infections, particularly denture stomatitis, is forthcoming. The use of plants in the synthesis of these alternative antimicrobials also means that a reduction in the use of antifungals is possible. This approach is welcomed, as antimicrobial resistance is on the increase.

Three different plants were selected for this study. Three different aqueous extracts of *B. lanuginose, H. cymosum,* and *S. crenata* were successfully used for the green synthesis of AgNPs. The biosynthesis protocol established in this study followed basic green protocols to formulate the intended NPs. Results showed that AgNPs were produced from the extracts. Different characterization techniques confirmed the formation of AgNPs. The effects of the three tested plant extracts on the particles’ morphology and crystallinity have been covered. The majority of the green-synthesized AgNP samples from *B. lanuginose* and *S. crenata* extracts were spherical in shape. The stability test demonstrated the stability of Bl.AgNPs and Hc.AgNPs with both tested media. This confirms the crucial role that *H. cymosum* and *B. lanuginose* extracts play as reducing and capping agents. The green-synthesized AgNPs exhibited promising antifungal activity that was both size- and concentration-dependent. AgNPs synthesized using *B. lanuginose* extracts (Bl.AgNPs) exhibited the most favorable antifungal results and satisfactory cytotoxic results. Furthermore, the cytotoxicity results showed that Bl.AgNP nanoparticles’ size produced the maximum cell death. The current study revealed the potential of the green approach in reducing the cytotoxicity of these samples. The synthesized AgNPs are applicable for different medical applications, including dental applications.

## Figures and Tables

**Table 1:** Zeta potential and size distribution of Bl.AgNPs, Hc.AgNPs, and Sc.AgNPs.

Sample Code	Bl.AgNPs	Hc.AgNPs	Sc.AgNPs
Zeta potential (mV)	−23.4	−18.8	−31.3
Hydrodynamic size (nm)	83.54	98.91	108.1

**Table 2:** OD readings for *C. albicans* at different treatment concentrations for the respective biosynthesized AgNP samples.

OD Average for 4 h				
Concentration (mg/mL)	Bl.AgNPs	Hc.AgNPs	Sc.AgNPs	Untreated
B (0.125)	0.037	0.033	0.040	0.229
C (0.062)	0.031	0.033	0.081	
D (0.031)	0.183	0.169	0.181	
E (0.015)	0.214	0.190	0.194	
F (0.007)	0.198	0.181	0.198	
G (0.003)	0.209	0.191	0.203	
H (0.0015)	0.237	0.224	0.215	

**Table 3:** Calculated concentrations of the different biosynthesized AgNPs, yielding MIC_50_ values at different time intervals.

	Calculated AgNP Concentrations		
Treatment	4 h	6 h	24 h
Sc.AgNPs	6.49	10.32	8.05
Bl.AgNPs	6.21	6.22	10.20
Hc.AgNPs	6.58	6.32	9.77

**Table 4:** Cell survival rates for green-biosynthesized AgNPs and their respective pure natural extracts.

	Average Percentage Cell Survival					
Conc. mg/mL	Bl.AgNPs	Hc.AgNPs	Sc.AgNPs	*B. lanuginose* ext.	*H. cymosum* ext.	*S. crenata* ext.
0.007	97.78	109.55	93.78	120.81	138.02	117.11
0.012	88.38	107.27	90.65	120.45	140.52	121.87
0.025	74.14	99.92	39.4	114.18	102.28	91.67
0.037	42.47	53.98	28.17	115.32	111.9	95.43
0.062	40.32	30.48	25.27	143.96	149.96	116.03

## Data Availability

The data available at (https://lib.uwc.ac.za/).
